# Prevalence and clinical relevance of digital ulcers in systemic sclerosis patients from the real-life: the experience of the SPRING Registry of the Italian Society for Rheumatology

**DOI:** 10.1007/s10067-025-07449-1

**Published:** 2025-05-15

**Authors:** Martina Orlandi, Giacomo De Luca, Clodoveo Ferri, Amelia Spinella, Federica Lumetti, Riccardo Cuoghi Costantini, Rossella De Angelis, Valeria Riccieri, Silvia Laura Bosello, Fabio Cacciapaglia, Veronica Codullo, Gianluigi Bajocchi, Corrado Campochiaro, Giovanni Zanframundo, Rosario Foti, Giovanna Cuomo, Alarico Ariani, Edoardo Rosato, Francesco Girelli, Elisabetta Zanatta, Ilaria Cavazzana, Francesca Ingegnoli, Maria De Santis, Giuseppe Murdaca, Giuseppina Abignano, Pettiti Giorgio, Alessandra Della Rossa, Maurizio Caminiti, Anna Maria Iuliano, Giovanni Ciano, Lorenzo Beretta, Gianluca Bagnato, Ennio Lubrano, Ilenia De Andres, Alessandro Giollo, Marta Saracco, Cecilia Agnes, Edoardo Cipolletta, Luca Magnani, Elisa Visalli, Carlo Iandoli, Antonietta Gigante, Greta Pellegrino, Erika Pigatto, Maria Grazia Lazzaroni, Franco Franceschini, Elena Generali, Gianna Mennillo, Simone Barsotti, Giuseppa Pagano Mariano, Federica Furini, Licia Vultaggio, Simone Parisi, Clara Lisa Peroni, Gerolamo Bianchi, Enrico Fusaro, Gian Domenico Sebastiani, Marcello Govoni, Salvatore D’Angelo, Franco Cozzi, Serena Guiducci, Andrea Doria, Carlo Salvarani, Florenzo Iannone, Lorenzo Dagna, Marco Matucci-Cerinic, Silvia Bellando-Randone, Dilia Giuggioli, SPRING-SIR (Systemic Sclerosis PRogression INvestiGation group of the Italian Society of Rheumatology) coworkers, Giorgio Amato, Alessia Benenati, Francesca Calabrese, Renato Carignola, Francesca Dall’Ara, Angelo De Cata, Marica Doveri, Nicoletta Romeo, Gianluca Sambataro, Rossella Talotta, Carlo Alberto Scirè, Gianpiero Landolfi, Davide Rozza, Greta Carrara, Anna Zanetti

**Affiliations:** 1 Department of Medical and Surgical Sciences for Children and Adults, University Hospital of Modena and Reggio Emilia School of Medicine, Via del Pozzo, 71-41125 Modena, Italy; 2 Unit of Immunology, Rheumatology, Allergy and Rare Diseases (UnIRAR), & Inflammation, Fibrosis and Aging Initiative (INFLAGE), IRCCS San Raffaele Scientific Institute, Milan, Italy; 3 Rheumatology Clinic ‘Madonna Dello Scoglio’ Cotronei, Crotone, Italy; 4 Rheumatology Unit, Department of Clinical and Molecular Sciences, Polytechnic University of Marche, Ancona, Italy; 5 Department of Internal Medicine, Anesthesiology and Cardiovascular Sciences, Sapienza University of Rome, Rome, Italy; 6 Rheumatology Division, Catholic University of the Sacred Heart, Fondazione Policlinico Universitario A. Gemelli-IRCCS, Rome, Italy; 7 Rheumatology Unit, Department of Precision and Regenerative Medicine-Ionian Area, University of Bari “Aldo Moro”, Bari, Italy; 8 Internal Medicine Service of Rheumatology “Miulli” General Hospital - Department of Medicine and Surgery, LUM “F. De Gennaro” University, Casamassima (Bari), Italy; 9 Department of Internal Medicine and Therapeutics, Università Di Pavia, Pavia, Italy; 10 Division of Rheumatology, Fondazione IRCCS Policlinico San Matteo, Pavia, Italy; 11 Rheumatology Unit, S. Maria Hospital-USL, IRCCS Institute, Reggio Emilia, Italy; 12 Rheumatology Unit, AOU Policlinico San Marco, Catania, Italy; 13 Department of Precision Medicine, Univeristy of Campania - Luigi Vanvitelli University, Naples, Italy; 14 Department of Medicine, Internal Medicine and Rheumatology, Azienda Ospedaliero Universitaria Di Parma, Parma, Italy; 15 Department of Translational and Precision Medicine, Sapienza University of Rome, Rome, Italy; 16 Department of Medicine, Rheumatology Unit, Ospedale GB Morgagni–L Pierantoni, Forlì, Italy; 17 Department of Rheumatology, University of Padua, Padua, Italy; 18 Rheumatology and Clinical Immunology, ASST Spedali Civili of Brescia; Department of Clinical and Experimental Sciences, University of Brescia, Brescia, Italy; 19 Division of Clinical Rheumatology, ASST Pini, Department of Clinical Sciences & Community Health, Research Center for Adult and Pediatric Rheumatic Diseases, Research Center for Environmental Health, Università Degli Studi Di Milano, Milan, Italy; 20 Department of Biomedical Sciences, Humanitas University, Pieve Emanuele-Milan and Research Hospital, Milan, Italy; 21 Department of Internal Medicine, University of Genoa and Allergology and Clinical Immunology Unit, Ospedale San Bartolomeo, Sarzana, Italy; 22 Department of Health Science, University of Basilicata. Rheumatology Unit, San Carlo Hospital, Potenza, Italy; 23 Rheumatology Unit ASO S. Croce E Carle Hospital, Cuneo, Italy; 24 Department of Rheumatology, University of Pisa, Pisa, Italy; 25 Departmental Rheumatology Unit, Grande Ospedale Metropolitano, Reggio Calabria, Italy; 26 Rheumatology Unit, San Camillo–Forlanini Hospital, Rome, Italy; 27 Local Health Department, Hospital of Ariano Irpino, Ariano Irpino, Italy; 28 Referral Center for Systemic Autoimmune Diseases, Fondazione IRCCS Ca’ Granda, Ospedale Maggiore Policlinico Di Milano, Milan, Italy; 29 Department of Clinical and Experimental Medicine, University of Messina, Messina, Italy; 30 Department of Rheumatology, University of Molise, Campobasso, Italy; 31 Rheumatology Unit, Azienda Ospedaliera Di Rilievo Nazionale Ed Alta Specializzazione Garibaldi, Catania, Italy; 32 Rheumatology Section, Department of Medicine, University of Verona, Verona, Italy; 33 Rheumatology Unit, Mauriziano-Umberto I Hospital, Turin, Italy; 34 Department of Medicine, Division of Rehabilitation, Torino, ASL TO5, Carmagnola, TO Italy; 35 IRCCS Ospedale Galeazzi Sant’Ambrogio, Milan, Italy; 36 Dipartimento Di Scienze Biomediche E Cliniche, Università Degli Studi Di Milano, Milan, Italy; 37 UOC Medicina Interna, Ospedale San Bassiano, Bassano del Grappa, Vicenza Italy; 38 Rheumatology Unit, Department of Medical Sciences, University of Ferrara and Azienda Ospedaliera-Universitaria S. Anna, Ferrara, Ferrara Italy; 39 Rheumatology Unit, Azienda Ospedaliera Universitaria Città Della Salute E Della Scienza Di Torino, Turin, Italy; 40 Rheumatology Unit, Department of Medical Specialities, Local Health Trust 3, Genoa, Italy; 41 Department of Medicine, Rheumatology Section, Villa Salus Hospital, Venice, Italy; 42 Department of Experimental and Clinical Medicine, Division of Rheumatology, University of Florence, Florence, Italy; 43 Vita Salute San Raffaele University, Milan, Italy

**Keywords:** Digital ulcers, Immunosuppressive therapy, Systemic sclerosis, Vascular disease

## Abstract

**Introduction:**

Digital ulcers (DU) are one of the most frequent manifestations in systemic sclerosis (SSc). The presence of DU seems to be a sentinel sign of internal organ involvement and is related to a poor prognosis of the disease. The aim of this study was to evaluate the prevalence and the relationship of DU with clinical manifestations/variants in a large SSc cohort from the SPRING registry.

**Methods:**

SSc patients fulfilling the ACR/EULAR 2013 classification criteria without missing data on digital ulcers were enrolled in a cross-sectional study. Logistic regression models were built to test the association between the presence of DU and SSc-related features.

**Results:**

Among 1873 eligible SSc patients, the presence of DU was significantly associated with gastrointestinal involvement (OR 1.88, 2.04 and 1.74; *p* < 0.001) and serum ATA positivity (OR 2.15; *p* < 0.001), as well as with telangiectasias, sclerodactyly, digital pitting scar, and calcinosis (OR 1.40, *p* = 0.005; OR 3.43, *p* < 0.001, OR 9.12, *p* < 0.001 and OR 2.77, *p* < 0.001; respectively). In the multivariable regression models, even after adjustment for covariates, ATA positivity (OR 1.76, *p* = 0.039), puffy fingers (OR 2.82, *p* < 0.001), and a higher revEUSTAR-AI (OR 6.63, *p* < 0.001) emerged as risk factors for the presence of DU. Moreover, a low presence of DU was recorded in SSc patients with a history of previous immunosuppressive treatments (OR 0.53, *p* = 0.032).

**Conclusion:**

In our Italian SSc cohort, DUs were significantly associated with the presence of puffy fingers, high revEUSTR-AI, and ATA seropositivity. Noteworthy, immunosuppressive treatments were associated with a low rate of DU, suggesting that they might contribute to the prevention of these harmful manifestations.

**Table:** 

**Key Points**
• *Digital ulcers were significantly associated with the presence of puffy fingers, high disease activity, and anti-Scl70 seropositivity.* • *Immunosuppressive treatments were associated with a low rate of digital ulcers, suggesting that they might contribute to the prevention of these harmful manifestations.*

**Supplementary Information:**

The online version contains supplementary material available at 10.1007/s10067-025-07449-1.

## Introduction

Digital ulcers (DU) are one of the most frequent manifestations in systemic sclerosis (SSc) [1–3], affecting around 58% of SSc patients [4], also in the very early phase of the disease [5]. Scleroderma vascular manifestations characterize the disease clinical course; they manifest as Raynaud Phenomenon (RP) since the prodromic phase of SSc, DU, pulmonary arterial hypertension (PAH), and/or scleroderma renal crisis (SRC) [6]. DUs represent an important burden both for the patient and the rheumatologist because they can complicate into local infection, osteomyelitis, gangrene, and eventually amputation [7–9]. Moreover, they are the major cause of pain and disability that may severely affect the patients’ quality of life [10–15]. Up to 66% of patients with DU can experience recurrent DU, despite optimal pharmacological therapies [4].

The pathophysiology of DU is complex and mainly remains in the scleroderma diffuse cutaneous microangiopathy and impaired neoangiogenesis [4, 6]; the skin sclerosis and joint contractures leading to increased susceptibility to trauma may severely contribute to vasculopathy and consequent difficult wound healing.

The skin ulcers that can be found in the course of SSc are quite different with regard to pathogenesis and clinico-therapeutic implications as a consequence of their specific localization [11]; among them, DUs are to be considered the direct expression of underlying scleroderma microangiopathy [2, 4]. Therefore, the presence of DU seems to be a sentinel sign of internal organ involvement [5] and has been related to a poor prognosis [16], regardless of the concurrence of PAH or SRC [17, 18].

In particular, there is a strong association between the presence and recurrence of DU and disease progression, as well as a shorter time to disease progression [19]. Finally, a history of DU recently emerged as a red flag for the replacement of myocardial fibrosis at cardiac magnetic resonance [20], likely due to the same vasculopathic pathogenesis for both complications.

The aim of the present study is to investigate the prevalence of DU in a large, multicentric SSc cohort of the Italian Society for Rheumatology SPRING registry (SIR-SPRING) and their correlation with other clinical features, disease activity, and ongoing treatments.

## Patients and methods

### Study population and data collection

This is a cross-sectional study performed on the national, multicentric SIR-SPRING; each of the 37 participating centers received approval from the local Ethical Committee, and all patients signed an informed consent. Patients enrolled in the SPRING registry were aged > 18 years, classified according to the SSc ACR/EULAR 2013 criteria [21]. Patients with missing data on the presence/absence of digital ulcers were excluded. DU were defined as previously reported [23] and classified as non-spontaneous healing “loss of substance” affecting one or more acral skin zones of the hands and feet [2, 24]. The diagnosis of the disease, as well as the nailfold videocapillaroscopy (NVC) and clinical evaluation, was made by an expert rheumatologist, as previously described [22]. Baseline information collected in the SPRING database included demographic data (covering geographical area and occupational data), autoantibody profile, clinical signs and symptoms of disease, disease cutaneous subset, organ involvement, previous and current therapies, categorized as vasoactive/vasodilating drugs (bosentan, ambrisentan, macitentan, sildenafil, tadalafil, iloprost, epoprostenol, PGE1, riociguat; calcium channel blocker), corticosteroids, and immunosuppression therapy (cyclophosphamide, methotrexate, mycophenolate, cyclosporine, rituximab, anti-tumor necrosis factor-α antagonists, tocilizumab, and abatacept).

In addition, the revised EUSTAR activity index (revEUSTAR-AI) was calculated to quantify SSc disease activity [25, 26]. The principal comorbidities, as defined in the Charlson comorbidity score and reported in our previous manuscript [27], were also considered.

## Statistical analysis

Demographic and disease-related features were analyzed through descriptive statistics results and were presented as numbers and percentages for categorical variables, otherwise as means (standard deviation) or medians (interquartile range) according to normal or non-normal distributions. Univariable and multivariable logistic regression models were estimated to test the association between the presence of DU and SSc-related characteristics. Multivariable models included covariates selected according to both their statistical significance and clinical relevance. A stepwise selection procedure based on the Akaike information criterion (AIC) was implemented in order to include in the final multivariable model the most informative variables among the candidate ones. Results were reported as Odds ratios (OR) with 95% confidence intervals (95% CI) and *p* values. Missing data are reported in tables and supplementary materials. The significance level alpha was set at 0.05. Imputation of missing data was not performed. As a result, for each variable, the estimation of the association with respect to DU was based only on the subjects without missing values in it. Moreover, the multivariable regression models were estimated from the data of the subjects without missing values in all the considered variables. All analyses were performed using R version 4.3.2 statistical software (The R Foundation For Statistical Computing, 2023, Vienna, Austria).

## Results

### Descriptive analysis

Based on the abovementioned inclusion criteria, 1873SSc patients were eligible for the study. In line with previous reports [25], two-thirds of patients were classified as limited cutaneous SSc (1252, 67.8%), with a mean age 59.4 ± 13.7 SD years and a mean disease duration of 8.85 ± 7.83 SD years. Anti-topoisomerase I antibodies (ATA) were positive in 640 patients (34.6%), while anti-centromere antibodies (ACA) in 563 patients (32.7%). DU were reported in 407 (22%) of patients. PAH was noted at right heart catheterization in 31 patients (1.8%), while SRC was reported in 22 (1.2%) cases. At baseline, 23.6% of the study population were on vasodilator/vasoactive treatment (at least one), and 14.8% were on immunosuppressive medications (at least one). The mean revEUSTAR-AI at baseline was 1.8 ± 1.3. A detailed description of the study population is reported in Table 1.

**Table 1:** Descriptive analysis of the population enrolled

Parameters	All pts (*n* = 1873)	Missing
Female sex, *n* (%)	1658 (88.7)	4
Age (years), mean (SD)	59.4 (13.7)	3
BMI, mean (SD)	24.1 (4.3)	215
Smoking habit, *n* (%)		202
Never smoker Past smoker Current smoker	1119 (67.0) 353 (21.1) 199 (11.9)	
Clinical features		
Disease duration (years), mean (SD)	8.85 (7.84)	23
Esophageal symptoms, *n* (%)	934 (50.0)	4
Gastric symptoms, *n* (%)	361 (19.3)	**7**
Intestinal symptoms, *n* (%)	366 (19.6)	5
Dyspnea (NYHA class ≥ 2), *n* (%)	510 (73.4)	1178
SSc sine scleroderma, *n* (%)	229 (12.4)	27
lcSSc, *n* (%)	1252 (67.8)	
dcSSc, *n* (%)	365 (19.8)	
Raynaud phenomenon, *n* (%)	1856 (99.2)	3
Puffy fingers *n* (%)	955 (51.1)	5
mRSS, mean (SD)	6.3 (6.9)	140
Sclerodactyly, *n* (%)	1313 (70.2)	2
Digital pitting scar, *n* (%)	880 (47.3)	0
Digital ulcers, *n* (%)	407 (21.7)	0
Gangrene, *n* (%)	10 (0.97)	6
Osteomyelitis, *n* (%)	12 (0.6)	7
Teleangiectasis, *n* (%)	1133 (60.6)	3
Calcinosis, *n* (%)	219 (11.7)	7
Muscle weakness, *n* (%)	267 (14.3)	6
Arthritis, *n* (%)	209 (11.2)	15
Tendon friction rubs, *n* (%)	156 (8.3)	5
SRC, *n* (%)	22 (1.2)	5
PAH, *n* (%)	31 (66.0)	1826
ILD, *n* (%)	1425 (76%)	0
DLCO (%), mean (SD)	68.7 (20.5)	532
FVC (%), mean (SD)	101.2 (22.7)	463
NVC Normal, *n* (%)	90 (4.9)	45
NVC Scleroderma Pattern Early, *n* (%)	365 (20.0)	
NVC Scleroderma Pattern Active, *n* (%)	797 (43.6)	
NVC Scleroderma Pattern Late, *n* (%)	434 (23.7)	
Megacapillariesi, *n* (%)	1175 (62.7)	0
Microhemorrhages, *n* (%)	733 (39.1)	0
Avascular areas, *n* (%)	519 (27.7)	0
Neoangiogenesis, *n* (%)	328 (17.5)	0
revEUSTAR-AI, mean (SD)	1.83 (1.33)	748
Laboratory profile		
ATA, *n* (%)	640 (34.6)	24
RNA pol3, *n* (%)	28 (1.9)	395
CENP, *n* (%)	563 (32.7)	150
Comorbidity		
Arterial hypertension, *n* (%)	449 (24.0)	0
Diabetes mellitus, *n* (%)	56 (3.0)	0
Complicated diabetes mellitus, *n* (%)	5 (0.3)	
Dyslipidemia, *n* (%)	208 (11.1)	0
Peripheral vascular disease, *n* (%)	103 (5.5)	0
Ongoing therapy		
Corticosteroids, *n* (%)	604 (58.4)	839
Vasodilator or vasoactive treatment (≥ 1), *n* (%)	443 (23.6)	0
Iloprost, *n* (%)	878 (73.5)	679
Antiplatelet/anticoagulant therapy, *n* (%)	76 (4.1)	0
Immunosuppressive treatment (≥ 1), *n* (%)	277 (14.8)	0
Past therapy		
Corticosteroids, *n* (%)	165 (16.0)	839
Vasodilator or vasoactive treatment (≥ 1), *n* (%)	1512 (80.3)	0
Iloprost, *n* (%)	137 (11.5)	679
Antiplatelet/anticoagulant therapy, *n* (%)	874 (46.7)	0
Immunosuppressive treatment (≥ 1), *n* (%)	506 (27.0)	0

**Legend**: *SD*, standard deviation; *BMI*, body mass index; *RP*, Raynaud’s phenomenon; *ATA*, anti-Scl-70 antibodies; *ARA*, anti-RNA polymerase III antibodies; *CENP-1*, anti-centromere antibodies; *SSc*, systemic sclerosis; *SSc sine scleroderma*, no skin involvement; *lcSSc*, limited skin involvement; *dcSSc*, diffuse skin involvement; *mRSS*, modified Rodnan Skin Score; *DPS*, digital pitting scar; *DU*, digital ulcers; *SRC*, scleroderma renal crisis; *NVC*, nailfold videocapillaroscopy; *TFR*, tendon friction rubs; *PAH*, pulmonary arterial hypertension; *ILD*, interstitial lung disease; *DLCO*, diffusing capacity of lung for carbon monoxide; *DLCO/VA*, diffusing capacity of lung for carbon monoxide divided by alveolar volume; *FVC*, forced vital capacity; *revEUSTAR-AI*, revised EUSTAR activity index

### Associations between digital ulcers and disease characteristics

Among 1873 SSc patients, the presence of DU was associated with ATA positivity (OR 2.15; *p* < 0.001) and GI involvement (OR 1.88, 2.04, and 1.74; *p* < 0.001; respectively for gastric, oesophageal, and intestinal involvement) at univariate analyses. Moreover, the presence of telangiectasias, sclerodactyly, digital pitting scar, and calcinosis were all positively associated with the presence of DU (OR 1.40, *p* = 0.005; OR 3.43, *p* < 0.001, OR 9.12, *p* < 0.001 and OR 2.77, *p* < 0.001; respectively). Differently, female sex and age were negatively associated with DU (OR 0.68, *p* = 0.021 and OR 0.98, *p* ≤ 0.001; respectively) as well as menopausal status (OR 0.69, *p* = 0.009), ACA positivity (OR 0.71, *p* = 0.008), and puffy fingers (OR 0.57, *p* ≤ 0.001). No significant association was found between ILD and DU.

Regarding the vasculopathic signs at NVC, the presence of a scleroderma pattern “active” at NVC was positively associated with the presence of DU (OR 1.24, *p* < 0.001), as well as the presence of avascular areas (OR 2.26, *p* < 0.001) and neoangiogenesis (OR 1.38, *p* < 0.021). A positive association was found between both the previous and ongoing vasodilator or vasoactive treatment (OR 2.49, *p* < 0.001 and OR 1.30, *p* = 0.038) as well as previous and ongoing immunosuppressive treatment (OR 1.30, *p* = 0.018 and OR 1.68, *p* < 0.001) and DU. No significant association was found regarding PAH and SRC (OR 1.77, *p* = 0.447). Detailed data are present in Table 2.

**Table 2:** Associations between digital ulcers and patients’ characteristics

Parameters	Patients, *n*	DU −	DU +	OR (95% CI)	*p* value
Female sex	1869	1310 (89.6%)	348 (85.5%)	0.68 (0.50–0.95)	**0.021**
Age	1870	60.03 (13.63)	56.97 (13.91)	0.98 (0.98–0.99)	** < 0.001**
BMI	1658	24.35 (4.34)	23.3 (4.35)	0.94 (0.91–0.97)	** < 0.001**
Smoking habit	1671				
Never smoker		885 (67.05%)	234 (66.67%)	Reference	
Past smoker		281 (21.29%)	72 (20.51%)	0.97 (0.72–1.30)	0.835
Current smoker		154 (11.67%)	45 (12.82%)	1.11 (0.77–1.59)	0.588
Menopausal status	1512	931 (76.44%)	203 (69.05%)	0.69 (0.52–0.91)	**0.009**
Laboratory					
ATA	1849	445 (30.71%)	195 (48.75%)	2.15 (0.93–6.05)	** < 0.001**
RNA pol3	1478	19 (1.64%)	9 (2.8%)	1.73 (0.77–3.86)	0.182
CENP	1723	461 (34.28%)	102 (26.98%)	0.71 (0.55–0.91)	**0.008**
Clinical characteristics					
Disease duration	1850	8.28 (7.52)	10.92 (8.58)	1.04 (1.03–1.05)	** < 0.001**
Esophageal symptoms	1869	683 (46.62%)	251 (62.13%)	1.88 (1.50–2.35)	** < 0.001**
Gastric symptoms	1866	244 (16.68%)	117 (29.03%)	2.04 (1.58–2.64)	** < 0.001**
Intestinal symptoms	1866	257 (17.55%)	109 (26.98%)	1.74 (1.34–2.25)	** < 0.001**
Dyspnea (NYHA class ≥ 2)	695	379 (71.92%)	131 (77.98%)	1.38 (0.92–2.09)	0.123
Skin involvement	1846				
SSc sine scleroderma		210 (14.52%)	19 (4.75%)	Reference	
lcSSc		1001 (69.23%)	251 (62.75%)	2.77 (1.70–4.52)	** < 0.001**
dcSSc		235 (16.25%)	130 (32.5%)	6.11 (3.65–10.24)	** < 0.001**
Raynaud phenomenon	1870	1452 (99.18%)	404 (99.51%)	1.67 (0.37–7.49)	0.503
Puffy fingers	1868	792 (54.1%)	163 (40.35%)	0.57 (0.46–0.72)	** < 0.001**
mRSS	1871	5.57 (6.42)	8.73 (7.76)	3.43 (2.53–4.66)	** < 0.001**
Sclerodactyly	1733	960 (65.57%)	353 (86.73%)	1.06 (1.04–1.08)	** < 0.001**
Digital pitting scar	1867	571 (38.95%)	335 (82.31%)	9.12 (6.85–12.16)	** < 0.001**
Gangrene	1867	4 (0.27%)	14 (3.47%)	13.14 (4.30–40.13)	** < 0.001**
Osteomyelitis	1866	3 (0.21%)	9 (2.23%)	11.12 (3.00–41.26)	** < 0.001**
Teleangiectasis	1870	889 (60.64%)	275 (67.57%)	1.40 (1.11–1.76)	**0.005**
Calcinosis	1866	132 (9.03%)	87 (21.53%)	2.77 (2.05–3.72)	** < 0.001**
Muscle weakness	1867	179 (12.24%)	88 (21.78%)	2.00 (1.50–2.65)	** < 0.001**
Arthritis	1858	144 (9.89%)	65 (16.17%)	1.76 (1.28–2.41)	** < 0.001**
Tendon friction rubs	1868	94 (6.42%)	62 (15.38%)	2.65 (1.88–3.73)	** < 0.001**
SRC	1868	15 (1.02%)	7 (1.73%)	1.70 (0.69–4.21)	0.248
PAH	47	22 (62.86%)	9 (75%)	1.77 (0.41–7.75)	0.447
ILD	1873	1110 (75.72%)	315 (77.4%)	1.10 (0.85–1.43)	0.4824
DLCO	1341	70.44 (20.1)	63.15 (20.72)	0.98 (0.98–0.99)	** < 0.001**
FVC	1410	103 (22.27)	95.01 (23.02)	0.98 (0.98–0.99)	** < 0.001**
Videocapillaroscopy					
NVC Normal	1828	325 (22.68%)	40 (10.13%)	Reference	0.23
NVC Scleroderma Pattern Early	1828	649 (45.29%)	148 (37.47%)	0.67 (0.35–1.29)	0.484
NVC Scleroderma Pattern Active	1828	278 (19.4%)	156 (39.49%)	1.24 (0.68–2.25)	** < 0.001**
NVC Scleroderma Pattern Late	1828	105 (7.33%)	37 (9.37%)	3.05 (1.67–5.57)	0.062
Megacapillariesi	1873	947 (64.6%)	228 (56.02%)	0.70 (0.56–0.87)	**0.002**
Microhemorrhages	1873	589 (40.18%)	144 (35.38%)	0.82 (0.65–1.02)	0.08
Avascular areas	1873	350 (23.87%)	169 (41.52%)	2.26 (1.80–2.85)	** < 0.001**
Neoangiogenesis	1873	241 (16.44%)	87 (21.38%)	1.38 (1.05–1.82)	**0.021**
revEUSTAR-AI	1126	1.34 (0.82)	3.39 (1.41)	5.24 (4.27–6.44)	** < 0.001**
Comorbidity					
Arterial hypertension	1873	366 (24.97%)	83 (20.39%)	0.77 (0.59–1.01)	0.056
Diabetes mellitus	1873	46 (3.14%)	10 (2.46%)	0.78 (0.39–1.55)	0.477
Complicated diabetes mellitus	1873	5 (0.34%)	0 (0%)	nc	nc
Dyslipidemia	1873	181 (12.35%)	27 (6.63%)	0.50 (0.33–0.77)	**0.001**
Peripheral vascular disease	1873	75 (5.12%)	28 (6.88%)	1.37 (0.87–2.15)	0.169
Ongoing therapy					
Corticosteroids	1034	452 (57.07%)	152 (62.81%)	0.92 (0.63–1.37)	0.695
Vasodilator or vasoactive treatment (≥ 1)	1873	331 (22.58%)	112 (27.52%)	1.30 (1.01–1.67)	0.038
Platelet aggregation inhibitors/oral anticoagulant	1873	54 (3.68%)	22 (5.41%)	1.49 (0.90–2.48)	0.122
Immunosuppressive treatment (≥ 1)	1873	194 (13.23%)	83 (20.39%)	1.68 (1.26–2.23)	** < 0.001**
Past therapy					
Corticosteroids	1034	121 (15.28%)	44 (18.18%)	1.09 (0.73–1.59)	0.695
Vasodilator or vasoactive treatment (≥ 1)	1873	1146 (78.17%)	366 (89.93%)	2.49 (1.76–3.52)	** < 0.001**
Platelet aggregation inhibitors/oral anticoagulant	1873	663 (45.23%)	211 (51.84%)	1.30 (1.05–1.62)	**0.018**
Immunosuppressive treatment (≥ 1)	1873	392 (26.74%)	114 (28.01%)	1.07 (0.83–1.36)	0.61

**Legend**: *SD*, standard deviation; *BMI*, body mass index; *RP*, Raynaud’s phenomenon; *ATA*, anti-Scl-70 antibodies; *ARA*, anti-RNA polymerase III antibodies; *CENP-1*, anti-centromere antibodies; *NYHA*, New York Heart Association; *SSc*, systemic sclerosis; *SSc sine scleroderma*, no skin involvement; *lcSSc*, limited skin involvement; *dcSSc*, diffuse skin involvement; *mRSS*, modified Rodnan Skin Score; *DPS*, digital pitting scar; *DU*, digital ulcers; *SRC*, scleroderma renal crisis; *NVC*, nailfold videocapillaroscopy; *TFR*, tendon friction rubs; *PAH*, pulmonary arterial hypertension; *ILD*, interstitial lung disease; *DLCO*, diffusing capacity of lung for carbon monoxide; *DLCO/VA*, diffusing capacity of lung for carbon monoxide divided by alveolar volume; *FVC*, forced vital capacity; *revEUSTAR-AI*, revised EUSTAR activity index. In bold type, the statistically significant associations (*p* < 0.05)

**Fig. 1:**
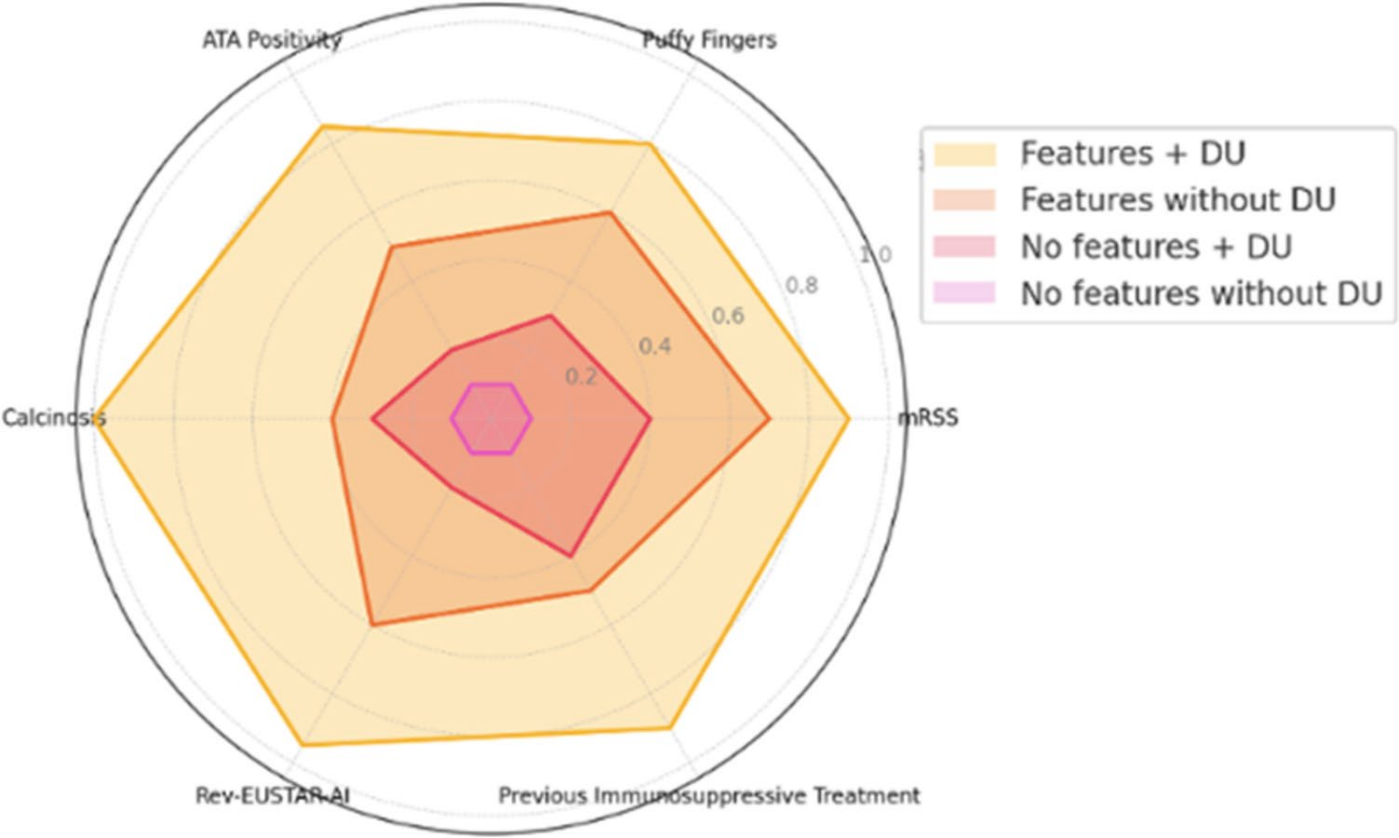
**Differences in disease burden between SSc phenotypes**. This spider plot illustrates the comparison of SSc phenotypes based on clinical features identified as significant in multivariate analysis. These features include mRSS, puffy fingers, ATA positivity, calcinosis, revEUSTAR-AI, and previous immunosuppressive treatment. Phenotypes and key insights: 1. **Clinical features + DU** (yellow area): This phenotype shows the highest intensity across most features, such as calcinosis, revEUSTAR-AI, and mRSS. Represents the most severe clinical phenotype, combining DU with systemic involvement. 2. **Clinical features without DU** (orange area): Moderate intensity in features like ATA positivity and puffy fingers. Indicates a milder systemic phenotype without DU. 3. **No clinical features + DU** (red area): Low intensity across all features but retains some involvement in features like revEUSTAR-AI. Suggests isolated vascular complications leading to DU without significant systemic manifestations. 4. **No clinical features without DU** (pink area): Minimal involvement in all features, representing the mildest phenotype with neither DU nor significant systemic disease

In multivariate models, the presence of DU was associated with ATA positivity (OR 1.76, CI 1.03–3.02, *p* = 0.039), the presence of puffy fingers (OR 2.82, CI = 1.66–4.86, *p* < 0.001), and a higher revEUSTAR-AI (OR 6.63, CI 5.05–8.92, *p* < 0.001) (Table 3). Conversely, a previous immunosuppressive treatment (OR 0.53, CI 0.29–0.94, *p* = 0.032) was the only feature associated with a lower presence of DU (OR 0.53, CI 0.29–0.94, *p* = 0.032) at multivariate analysis. These associations are also reported in Fig. 1.

**Table 3:** Multivariable logistic regression model to predict the presence of digital ulcers

Parameters	OR (lower limit IC-upper limit IC)	*p* value
Female sex	1.00 (0.51–2.04)	0.991
Age	0.99 (0.97–1.00)	0.126
mRSS	0.89 (0.85–0.93)	** < 0.001**
Disease duration	1.02 (0.99–1.05)	0.266
ATA	1.76 (1.03–3.02)	**0.039**
SSc sine scleroderma	Reference	
lcSSc	2.72 (0.97–9.17)	0.075
dcSSc	3.17 (0.92–12.48)	0.080
Puffy fingers	2.82 (1.66–4.86)	** < 0.001**
Sclerodactyly	1.19 (0.68–2.11)	0.552
Calcinosis	2.68 (1.40–5.19)	**0.003**
Teleangiectasis	0.62 (0.36–1.06)	0.082
Esophageal symptoms	0.84 (0.49–1.41)	0.507
Gastric symptoms	1.32 (0.72–2.42)	0.365
Intestinal symptoms	0.92 (0.49–1.71)	0.804
NVC Scleroderma Pattern Early	0.70 (0.22–2.40)	0.565
NVC Scleroderma Pattern Active	0.78 (0.26–2.51)	0.672
NVC Scleroderma Pattern Late	2.11 (0.62–7.62)	0.240
Megacapillaries	0.64 (0.35–1.17)	0.143
Microhemorrhages	0.86 (0.51–1.44)	0.563
Avascular areas	0.85 (0.41–1.75)	0.666
Neoangiogenesis	1.33 (0.73–2.44)	0.351
revEUSTAR-AI	6.63 (5.05–8.92)	** < 0.001**
Previous immunosuppressive treatment (≥ 1)	0.53 (0.29–0.94)	**0.032**
Previous vasodilator or vasoactive treatment (≥ 1)	1.57 (0.71–3.55)	0.276
Previous platelet aggregation inhibitors/oral anticoagulant	1.37 (0.85–2.19)	0.193
Previous calcium channel blocker	0.80 (0.47–1.36)	0.409

**Legend**: *ATA*, anti-Scl-70 antibodies; *NVC*, nailfold videocapillaroscopy; *lcSSc*, limited skin involvement; *dcSSc*, diffuse skin involvement; *mRSS*, modified Rodnan Skin Score; *revEUSTAR-AI*, revised EUSTAR activity index. In bold type, the statistically significant associations (*p* < 0.05)

## Discussion

Our multicenter study examined the prevalence and clinical relevance of DU in the largest cohort of Italian SSc patients by analyzing data from the SIR-SPRING registry. We found that 22% of patients reported the presence of DU, consistent with previous studies indicating a similar prevalence across different populations ranging from 10 to 40% [3, 4, 10, 28]. Moreover, a positive association with some clinico-serological findings of the disease was observed, mainly regarding the peripheral microvascular involvement (RP, telangiectasis, calcinosis, major capillaroscopic alteration and DPS), sclerodactyly, serum ATA positivity, and GI involvement. Moreover, DU were associated with disease duration and disease activity. The strong positive association of DU with specific clinical signs such as sclerodactyly, telangiectasias, DPS, and calcinosis may identify SSc variants with more extensive vascular involvement. The pathophysiology of DU in SSc is complex and involves several mechanisms, in which vascular diseases play a central role. The key pathophysiological factors driving vascular involvement in SSc-DU are believed to be ischemia, vasculopathy, platelet activation and fibrin deposition, whereas DU could also be linked to mechanical stress, as a result of recurrent microtrauma and increased skin tension. [2, 3]. Moreover, both circulating endothelial progenitor cells with weakened angiogenic function and regression of capillaries and small vessels commonly observed in SSc patients strongly suggest an impaired activity of endothelial cells in the microvascular homeostasis, although the specific mechanisms have not yet been elucidated [29]. In fact, histologic evaluation of digital arteries from patients with SSc demonstrates intimal hyperplasia, adventitial fibrosis, resulting in greater than 75% luminal narrowing and intraluminal thrombosis [30] In clinical practice, the presence of this symptomatic complex, along with ATA seropositivity, may be highly predictive of DU development and may drive more adequate preventive measures and treatment regimens [3, 15, 31]. Moreover, alongside these clinical aspects, another interesting aspect of our study regards the significant correlation between DUs and major vasculopathic signs observed at NVC, i.e., active scleroderma pattern, avascular areas, and/or neoangiogenesis, which are the expression of severe scleroderma microvasculopathy [3, 32]. This reinforces the understanding of DU as manifestations of the underlying microvascular dysfunction characteristic of SSc [33]. In fact, the pathophysiology of DU is mainly referred to the scleroderma diffuse cutaneous microangiopathy, and impaired neoangiogenesis. Prior research has demonstrated that abnormalities in capillary morphology can serve as predictive markers for the development of DUs, thereby emphasizing the utility of NVC in clinical assessments and as monitoring tool even after Ssc diagnosis, to better identify SSc patients at higher risk of DU [34].The well-documented association of DU with serum ATA [35, 36] reinforces the prognostic value of these clinical and serological parameters, hallmarks of more aggressive disease phenotypes [3, 15, 31]. Moreover, also the association of DU with gastrointestinal involvement is largely reported [3, 10, 15, 35], asmodifications of blood flow have been observed both in the stomach and in the entire intestine [37, 38]. In fact, the involvement of the circulation of this tract may be correlated to motility impairment, which is the main cause of stasis, bowel dilatation with bacterial overgrowth, malabsorption, weight loss and a decreased survival rate [39]. In the multivariate analysis, ATA positivity together with puffy hands and calcinosis remains significantly associated with the presence of DU. The analysis of our patient’s cohort revealed important information regarding the use of intravenously (IV) applied prostanoids such as iloprost that, in our cohort, is present in over 70% of patients. This data is quite in contrast with previous data from other centers. Moinzadeh et al., for example, reported that in SSc patients from German Network for SSc registry, only 21% of patients were treated with IV prostanoids [40]. These data suggest that, worldwide, many patients with SSc with signs of peripheral vasculopathy do not yet receive sufficient vasoactive treatment, but it must be considered that in some countries (such as in North America), this treatment is not approved. Moreover, in our study, both previous and ongoing vasodilator drugs, mainly intravenous iloprost, were associated with the presence of DU at univariate analysis. This observation suggests that vasoactive treatments may usefully affect the SSc vascular complications, but they may not be sufficient to prevent DU development. This is consistent with literature indicating that a substantial percentage of patients with DU continue to experience recurrent episodes despite optimal medical therapy [4, 10]. Anyway, this association was not confirmed at the multivariate analysis. In contrast, in multivariate analysis previous IV vasodilating therapy remains significantly associated with DU, suggesting a possible role in preventing the DU onset through the reduction of vascular impairment. Noteworthy, previous immunosuppressive treatments emerged as protective factors for the presence of DU at multivariate analysis. This finding could indirectly support the notion that immune-mediated inflammation could contribute to the pathogenesis of vasculopathic DU. Overall, a possible preventive role of both vasoactive and immunosuppressive treatments on the development of SSc-DU seems to be highlighted at least in part by the significant reduction of DU prevalence recently observed in older SSc patients’ populations (from 54 to 16.5%) [28]. The knowledge of SSc pathogenesis has significantly advanced in the last three decades, along with improved outcome and the introduction of novel pathogenetic treatments, often at the early stages of the disease, as well as to improved diagnostic tools, allow to observe individuals with milder clinical variants [28]. Conversely, we found no significant association between DU and the incidence of PAH or SRC, probably due to the low number of patients with these manifestations in our SPRING cohort. This finding aligns with some studies that have also failed to demonstrate a direct association between these complications and DU [5]. Despite the robustness of our findings, several limitations must be acknowledged. The retrospective design may introduce biases related to data collection and patient selection. The reliance on registry data means that not all clinical variables were uniformly captured, which could affect the comprehensiveness of our analysis. Moreover, the cross-sectional nature of the study limits our ability to draw causal inferences.

In conclusion, our data underlines some important associations between the presence of DU and other scleroderma clinic-serological features, as well as the preventive role of previous immunosuppressive treatments on DU development. In the near future, in-depth multicentric-longitudinal studies may better clarify the role of preventive/therapeutic options for DU SSc manifestations.

## Supplementary Information

Below is the link to the electronic supplementary material.

Supplementary file1 (DOCX 33 KB)

## Data Availability

Data available on request from the corresponding Author MO.
