# Supplementary material for: Prevalence and clinical relevance of digital ulcers in systemic sclerosis patients from the real-life: the experience of the SPRING Registry of the Italian Society for Rheumatology
Source: Clin Rheumatol. 2025 May 15;44(7):2849–60. doi: 10.1007/s10067-025-07449-1 (PMC12234588; doi:10.1007/s10067-025-07449-1)
Supplement: Supplementary file 1 — Supplementary file1 (DOCX 33 KB) [file 10067_2025_7449_MOESM1_ESM.docx]

**Supplementary material**

**Title**

**Prevalence and clinical relevance of digital ulcers in systemic sclerosis patients from the real-life: the experience of the SPRING Registry of the Italian Society for Rheumatology.**

Martina Orlandi^1^, Giacomo De Luca^2^, Clodoveo Ferri^1,3^, Amelia Spinella^1^, Federica Lumetti^1^, Riccardo Cuoghi Costantini^1^, Rossella De Angelis^4^, Valeria Riccieri^5^, Silvia Laura Bosello^6^, Fabio Cacciapaglia^7,8^, Veronica Codullo^9^, Gianluigi Bajocchi^10^, Corrado Campochiaro^2^, Giovanni Zanframundo^9^, Rosario Foti^11^, Giovanna Cuomo^12^, Alarico Ariani^13^, Edoardo Rosato^14^, Francesco Girelli^15^, Elisabetta Zanatta^16^, Ilaria Cavazzana^17^, Francesca Ingegnoli^18^, Maria De Santis^19^, Giuseppe Murdaca^20^, Giuseppina Abignano^21^, Pettiti Giorgio^22^, Alessandra Della Rossa^23^, Maurizio Caminiti^24^, Anna Maria Iuliano^25^, Giovanni Ciano ^26^, Lorenzo Beretta^27^, Gianluca Bagnato^28^, Ennio Lubrano^29^, Ilenia De Andres^30^, Alessandro Giollo^31^, Marta Saracco^32^, Cecilia Agnes^33^, Edoardo Cipolletta^4^, Luca Magnani^8^, Elisa Visalli^10^, Carlo Iandoli^11^, Antonietta Gigante^13^, Greta Pellegrino^34,35^, Erika Pigatto^36^, Maria Grazia Lazzaroni^17^, Franco Franceschini^17^, Elena Generali^19^, Gianna Mennillo ^21^, Simone Barsotti^23^, Giuseppa Pagano Mariano^24^, Federica Furini^37^, Licia Vultaggio^37^, Simone Parisi^38^, Clara Lisa Peroni^38^, Gerolamo Bianchi^39^, Enrico Fusaro^38^, Gian Domenico Sebastiani^25^, Marcello Govoni^37^, Salvatore D'Angelo^21^, Franco Cozzi^40^, Serena Guiducci^41^, Andrea Doria^34^, Carlo Salvarani^9^, Florenzo Iannone^7^, Lorenzo Dagna^2,42^, Marco Matucci-Cerinic^2,42^, Silvia Bellando-Randone^41^, Dilia Giuggioli^1^ and SPRING-SIR (Systemic Sclerosis PRogression INvestiGation group of the Italian Society of Rheumatology) coworkers.

**Corresponding Author**

Martina Orlandi, MD, PhD, Assistant Professor,

University Hospital of Modena and Reggio Emilia School of Medicine, -.

via del Pozzo 71-41125 Modena, Italy.

+390594225777

martina.orlandi@unimore.it

**Affiliations**

^1^ University Hospital of Modena and Reggio Emilia School of Medicine, Department of Medical and Surgical Sciences for Children and Adults, Modena, Italy;

^2^ Unit of Immunology, Rheumatology, Allergy and Rare Diseases (UnIRAR), & Inflammation, Fibrosis and aging initiative (INFLAGE), IRCCS San Raffaele Scientific Institute, Milan, Italy

^3^ Rheumatology Clinic ‘Madonna dello Scoglio’ Cotronei, Crotone, Italy;

^4^ Rheumatology Unit, Department of Clinical and Molecular Sciences, Polytechnic University of Marche, Ancona, Italy;

^5^ Department of Internal Medicine, Anesthesiology and Cardiovascular Sciences, Sapienza University of Rome, Rome, Italy;

^6^ Rheumatology Division, Catholic University of the Sacred Heart, Fondazione Policlinico Universitario A. Gemelli- IRCCS – Rome, Italy;

^7^ Rheumatology Unit, Department of Precision and Regenerative Medicine-Ionian Area, University of Bari "Aldo Moro", Bari, Italy;

^8^ Internal Medicine Service of Rheumatology "Miulli" General Hospital - Department of Medicine and Surgery LUM "F. De Gennaro" University, Casamassima (Bari), Italy;

^9^ Department of Internal Medicine and Therapeutics, Università di Pavia, Italy; Division of Rheumatology, Fondazione IRCCS Policlinico San Matteo, Pavia, Italy

^10^ Rheumatology Unit, S. Maria Hospital-USL, IRCCS Institute, Reggio Emilia, Italy

^11^ Rheumatology Unit, AOU Policlinico San Marco, Catania, Italy.

^12^Department of Precision Medicine - Univeristy of Campania - Luigi Vanvitelli University, Naples, Italy;

^13^ Department of Medicine, Internal Medicine and Rheumatology, Azienda Ospedaliero Universitaria di Parma, Parma, Italy;

^14^ Department of Translational and Precision Medicine, Sapienza University of Rome, Rome, Italy;

^15^ Department of Medicine, Rheumatology Unit, Ospedale GB Morgagni–L Pierantoni, Forlì, Italy;

^16^ Department of Rheumatology, University of Padua, Padova, Italy;

^17^   Rheumatology and Clinical Immunology, ASST Spedali Civili of Brescia; Department of Clinical and Experimental Sciences, University of Brescia, Brescia, Italy;

^18^ Division of Clinical Rheumatology, ASST Pini, Dept. of Clinical Sciences & Community Health, Research Center for Adult and Pediatric Rheumatic Diseases, Research Center for Environmental Health, Università degli Studi di Milano, Milan, Italy;

^19^ Department of Biomedical Sciences, Humanitas University, Pieve Emanuele-Milan and Research Hospital, Milan, Italy;

^20^ Department of Internal Medicine, University of Genoa and allergology and clinical immunology unit, ospedale San Bartolomeo Sarzana, Italy;

^21^ Department of Health Science, University of Basilicata. Rheumatology Unit, San Carlo Hospital, Potenza, Italy,

^22^ Rheumatology Unit ASO S. Croce e Carle Hospital, Cuneo, Italy;

^23^ Department of Rheumatology, University of Pisa, Pisa, Italy;

^24^ Departmental Rheumatology Unit, Grande Ospedale Metropolitano, Reggio Calabria, Italy

^25^ Rheumatology Unit, San Camillo–Forlanini Hospital, Rome, Italy;

^26^ Hospital of Ariano Irpino, Local Health Department, Ariano Irpino, Italy;

^27^ Referral Center for Systemic Autoimmune Diseases, Fondazione IRCCS Ca’ Granda, Ospedale Maggiore Policlinico di Milano, Milan, Italy;

^28^ Department of Clinical and Experimental Medicine, University of Messina, Messina, Italy;

^29^ Department of Rheumatology, University of Molise, Campobasso, Italy;

^30^ Rheumatology Unit, Azienda Ospedaliera di Rilievo Nazionale ed Alta Specializzazione Garibaldi, Catania, Italy;

^31^ Rheumatology Section, Department of Medicine, University of Verona,Verona, Italy;

^32^ Rheumatology Unit, Mauriziano-Umberto I Hospital, Turin, Italy;

^33^ Department of Medicine, Division of Rehabilitation, Torino, ASL TO5, Carmagnola (TO), Italy;

^34^ IRCCS Ospedale Galeazzi Sant’Ambrogio, Milano

^35^Dipartimento di Scienze Biomediche e Cliniche, Università degli Studi di Milano, Milano

^36^ UOC Medicina Interna, Ospedale San Bassiano, Bassano del Grappa, Vicenza, Italy

^37^ Rheumatology Unit, Department of Medical Sciences, University of Ferrara and Azienda Ospedaliera-Universitaria S. Anna, Ferrara, Ferrara, Italy;

^38^ Rheumatology Unit, Azienda Ospedaliera Universitaria Città Della Salute e della Scienza di Torino, Turin, Italy;

^39^ Rheumatology Unit, Department of Medical Specialities, Local Health Trust 3, Genoa, Italy;

^40^ Department of Medicine, Rheumatology Section, Villa Salus Hospital, Venice, Italy.

^41^ Department of Experimental and Clinical Medicine, Division of Rheumatology, University of Florence, Florence, Italy.

^42^ Vita Salute San Raffaele University, Milano, Italy

**Supplementary Table 1.** **Descriptive Analysis of the population enrolled; data not included in Table 1 in the manuscript.**

|  | **All patients** | |
| --- | --- | --- |
| **Parameters** | All pts1873 | Missing |
| **Demographic** | |  |
| Ethnicity, n (%)  Caucasian  Afro-American  Asian  Other | \| 1782 (98.5) \| \| --- \| \| 9 (0.5) \| \| 5 (0.3) \| \| 14 (0.8) \| | 63 |
| Work occupation, n (%)  Full time  Not full time  Retired  Unemployed | 551 (34.3)  97 (6.0)  610 (38.0)  349 (21.7) | 266 |
| Manual work, n (%) | \| 239 (38.2) \| \| --- \| | 1238 |
| Toxic exposition, n (%) | 57 (3.4) | 183 |
| Previous pregnancy, n (%) | 1629 (87) | 0 |
| Menopausal status, n (%) | 1134 (75) | 361 |
| Previous Miscarriage, n (%) | 270 (21.5) | 617 |
| **Clinical features** | |  |
| Resting sPAP (mmHg) | 22.8 (16.5) | 325 |
| ESR (mm/h), mean (SD) | 21.7 (17.7) | 245 |
| RPC (mg/L), mean (SD) | 9.3 (25.0) | 185 |
| **Laboratory profile** | | |
| ANA, n (%) | 1800 (97.4) | 26 |
| ANCA, n (%) | 24 (1.5) | 302 |
| Anti-dsDNA, n (%) | 18 (1.1) | 275 |
| ACLA/anti β2GPI/LAC, n (%) | 68 (4.3) | 296 |
| **Comorbidity** | |  |
| Myocardial infarction, n (%)  Heart failure, n (%) | 54 (2.9)  39 (2.1) | 0  0 |
| Cerebrovascular disease, n (%)  Ictus, n (%)  hemiplegia, n (%) | 34 (1.8)  17 (0.9)  4 (0.2) | 0  0  0 |
| Chronic pulmonary disease (not ILD), n (%) | 81 (4.3) | 0 |
| Kidney disease (moderate to severe), n (%) | 48 (2.6) | 0 |
| Liver disease (mild), n (%)  Liver disease (moderate to severe), n (%) | 57 (3.0)  24 (1.3) | 0 |
| Cancer, n (%)  Metastatic cancer, n (%)  Leukemia, n (%)  Lymphoma, n (%) | 138 (7.4)  8 (0.4)  2 (0.11)  6 (0.3) | 0 |
| Dementia, n (%) | 4 (0.2) | 0 |
| Osteoporosis, n (%) | 245 (13.1) | 0 |
| Gastroduodenal ulcer, n (%) | 49 (2.6) | 0 |
| **Ongoing Therapy** |  |  |
| Bosentan, n (%) | 371 (45.3) | 1054 |
| Ambrisentan, n (%) | 11 (2.6) | 1450 |
| Macitentan, n (%) | 38 (8.5) | 1424 |
| Sildenafil, n (%) | 59 (12.3) | 1395 |
| Tadalafil, n (%) | 13 (3.7) | 1437 |
| epoprostenolo, n (%) | 3 (0.7) | 1465 |
| PGE1, n (%) | 41 (8.7) | 1403 |
| Riociguat, n (%) | 1 (0.2) | 1456 |
| Calcium channel blocker | 239 (12.76) | 0 |
| Beta blocker, n (%) | 133 (25) | 1341 |
| Antiplatet therapy, n (%) | 830 (73.3) | 741 |
| anticoagulant therapy, n (%) | 53 (11.6) | 1418 |
| Cyclophosphamide, n (%) | 14 (2.4) | 1290 |
| Micophenolate, n (%) | 228 (36.7) | 12511 |
| Methotrexate, n (%) | 158 (24.9) | 1238 |
| Rituximab, n (%) | 41 (8.6) | 1395 |
| Anti-TNF, n (%) | 1 (0.2) | 1445 |
| Tocilizumab, n (%) | 14 (3.2) | 1436 |
| Abatacept, n (%) | 4 (0.9) | 14441 |
| **Past therapy** |  |  |
| Bosentan, n (%) | 112 (13.7) | 1054 |
| Ambrisentan, n (%) | 2 (0.5) | 1450 |
| Macitentan, n (%) | 2 (0.4) | 1424 |
| Sildenafil, n (%) | 19 (4.0) | 1395 |
| Tadalafil, n (%) | 7 (1.6) | 1437 |
| epoprostenolo, n (%) |  | 1465 |
| PGE1, n (%) | 41 (8.7) | 1403 |
| Riociguat, n (%) | 1 (0.1) | 416 |
| Calcium channel blocker | 994 (53.07) | 0 |
| Beta blocker, n (%) | 11 (2.1) | 1341 |
| Antiplatet therapy, n (%) | 69 (6.1) | 741 |
| anticoagulant therapy, n (%) | 7 (1.5) | 1418 |
| Cyclophosphamide, n (%) | 185(31.7) | 1290 |
| Micophenolate, n (%) | 41 (6.6) | 251 |
| Methotrexate, n (%) | 123 (19.4) | 1238 |
| Rituximab, n (%) | 32 (6.7) | 1395 |
| Anti-TNF, n (%) | 9 (2.1) | 1445 |
| Tocilizumab, n (%) | 5 (1.1) | 1436 |
| Abatacept, n (%) | 5 (1.2) | 1444 |

***Legend****: SD: Standard deviation; ANA: Antinuclear Antibodies; ANCA:* *Anti-neutrophil cytoplasmic antibody; Beta-2-Glycoprotein I Antibodies; ACLA: Anti-Cardiolipin antibody; LAC: Lupus anticoagulant; ESR: erythrocyte sedimentation rate; CRP: C-reactive protein; NYHA: New York Heart Association; sPAP: systolic pulmonary artery pressure; LVEF: Left ventricular ejection fraction;* *PGE1: Prostaglandin E1; PDE5i: phosphodiesterase 5 inhibitors, ERA: endothelin receptor antagonists; TNF: tumor necrosis factor.*

**Supplementary Table 2. Correlation between digital ulcers and disease characteristics; data not included in Table 1 in the manuscript.**

| **Parameters** | **OR (Lower limit IC- Upper limit IC)** | | **p value** |
| --- | --- | --- | --- |
| Smoking habit  Never smoker  Past smoker  Current smoker | Reference  0.97 (0.72-1.30)  1.11 (0.77-1.59) | | 0.835  0.588 |
| Ethnicity  Caucasian  Afro-American  Asian  Other | Reference  0.45 (0.06-3.58)  0.89 (0.10-8.01)  0.97 (0.27-3.51) | | 0.447  0.919  0.967 |
| Work occupation  Full time  Not full time  Retired  Unemployed | Reference  0.81 (0.48-1.36)  0.56 (0.42-0.74)  0.80 (0.58-1.11) | | 0.430  **<0.001**  0.180 |
| Manual work | 1,00 (0,69-1,45) | | 0,994 |
| Toxic exposition | 0.89 (0.45-1.73) | | 0.723 |
| Previous pregnancy | 0.90 (0.26-3.15) | | 0.869 |
| Previous Miscarriage | 1.05 (0.97-1.14) | | 0.235 |
| **Laboratory** |  | |  |
| ANA, n (%) | 2.38 (0.93-6.05) | | 0.069 |
| ANCA, n (%) | 1.48 (0.61-3.61) | | 0.384 |
| Anti-dsDNA, n (%) | 2.92 (1.14-7.45) | | **0.025** |
| ACLA/anti β2GPI/LAC, n (%) | 1.10 (0.62-1.94) | | 0.756 |
| **Clinical characteristics** |  | |  |
| Resting sPAP (mmHg), mean (SD) | 1.01 (1.00-1.01) | | 0.156 |
| ESR (mm/h), mean (SD) | 1.00 (0.99-1.01) | | 0.991 |
| RPC (mg/L), mean (SD) | 1.01 (1.00-1.02) | | 0.213 |
| **Comorbidity** |  | |  |
| Myocardial infarction  Heart failure, n (%) | 1.68 (0.94-3.02)  1.25 (0.60-2.58) | | 0.081  0.550 |
| Cerebrovascular disease, n (%)  Ictus  hemiplegia | 1,11 (0.50-2.47)  1.11 (0.36-3.42)  1.20 (0.12-11.58) | | 0,797  0.857  0.874 |
| Chronic pulmonary disease (not ILD), n (%) | 0.95 (0.55-1.65) | | 0.868 |
| Kidney disease (moderate to severe) , n (%) | 2.21 (1.22-4.02) | | **0.009** |
| Liver disease (mild), n (%)  Liver disease (moderate to severe), n (%) | 0.76 (0.38-1.52)  0.32 (0.08-1.38) | | 0.438  0.128 |
| Cancer, n (%)  Metastatic cancer  Leukemia  Lymphoma | 1.00 (0.66-1.52)  2.17 (0.52-9.12)  3.61 (0.23-57.81)  1.80 (0.33-9.89) | | 0.998  0.290  0.365  0.496 |
| Dementia, n (%) | 1.20 (0.12-11.58) | | 0.874 |
| Osteoporosis, n (%) | 1.29 (0.95-1.76) | | 0.105 |
| Gastroduodenal ulcer, n (%) | 0.59 (0.26-1.33) | | 0.205 |
| **Ongoing Therapy** |  | |  |
| Calcium channel blocker, n (%) | 1.34 (0.98-1.83) | | 0.069 |
| Beta blocker, n (%) | 0.45 (0.13-1.57) | | 0.208 |
| Cyclophosphamide, n (%) | 0.77 (0.17-3.58) | | 0.738 |
| Micophenolate, n (%) | 2.00 (1.05-3,83) | | **0.036** |
| Methotrexate, n (%) | 1.41 (0.93-2.14) | | 0.102 |
| Rituximab, n (%) | 1.86 (0.89-3.91) | | 0.099 |
| Anti-TNF, n (%) | 2.78 (0.74-10.41) | | 0.129 |
| Tocilizumab, n (%) | 2.32 (0.39-13.92) | | 0.359 |
| Abatacept, n (%) | 1.15 (0.12-11.12) | | 0.902 |
| **Previous Therapy** |  | |  |
| Cyclophosphamide, n (%) | 0.82 (0.51-1.33) | | 0.421 |
| Micophenolate, n (%) | 0.74 (0.51-1.06) | | 0.104 |
| Methotrexate, n (%) | 1.20 (0.82-1.77) | | 0.342 |
| Rituximab, n (%) | 1.65 (0.85-3.23) | | 0.142 |
| Anti-TNF, n (%) | nc | | nc |
| Tocilizumab, n (%) | 1.39 (0.43-4.46) | | 0.580 |
| Abatacept, n (%) | 2.31 (0.38-13.86) | | 0.361 |
|  |  |  |  |

***Legend****: SD: Standard deviation; ANA: Antinuclear Antibodies; ANCA:* *Anti-neutrophil cytoplasmic antibody; Beta-2-Glycoprotein I Antibodies; ACLA: Anti-Cardiolipin antibody; LAC: Lupus anticoagulant; ESR: erythrocyte sedimentation rate; CRP: C-reactive protein; NYHA: New York Heart Association; sPAP: systolic pulmonary artery pressure; LVEF: Left ventricular ejection fraction, nc: not calculable.*
